# Dr. Benjamin F. Showerman

**Published:** 1905-07

**Authors:** 


Dr. Benjamin F. Showerman, of Batavia, N. Y., died at his home June 15, 1905, aged 41 years. His disease was cancer of the stomach of which he had been a long time sufferer. He received his preliminary education at Batavia and graduated in medicine at the University-Bellevue medical college in 1886. Dr. Showerman began practice at once in Batavia and pursued his professional occupation unintermittingly until his last illness. He was a member of the various medical societies and of the board of pension examiners of that district. Dr. Showerman was married in 1893 to Minnie, daughter of the late William C. Simpson. His wife died in 1898. He is survived by his mother and three sisters, Miss Jennie Showerman, of Buffalo, Mrs. Ralph Gillette, and Mrs. William E. Webster, of Batavia.

